# Casting Clasp and Saddle in One Piece

**Published:** 1920-01

**Authors:** F. E. Roach


### CASTING CLASP AND SADDLE IN ONE PIECE

BY F. E. ROACH, D.D.S.

Take these impressions in plaster of Paris, or complaster, with the ordinary crown and bridge tray that includes all that portion of the arch to be utilized, remove the tray and make a longitudinal or mesial-distal incision centrally, and split the impression and remove the two halves from the mouth. You can do that and get reasonably good results.

The objection to that plan of taking the impression is that in making this incision on the occlusal surface you are very likely to mutilate the occlusal surface of the space adjacent to the space where it is most desirable to place your occlusal rest. It is very important to my mind that we have an accurate impression of the occlusal surface and the surface of that tooth next to the space adjacent to the occlusal surface. We are not very particular about the under-cuts at the cervical and proximal angles, but I would rather have always, in all of this work, an exact impression of these teeth. I like to have in my model an exact reproduction of the conditions in the mouth where I can see just what I am dealing with, as a bicuspid and molar space.

If I get an accurate impression of the teeth and the space I can more intelligently design my appliance. I can design the outline of the clasp according to the contour of the tooth and I can determine just how much filing of the under-cut is necessary to facilitate the removal and placement of the appliance. It is absolutely necessary to eliminate the mesial and distal under-cuts. You can not have these appliances so that the casting extends in around the under-cuts as you can not get them on or off. It is a short space and your appliance is very rigid and there is no chance to tip it or spring the teeth to get it in or out. It must go in mechanically straight up and down.

If you have a tooth on one side and one on the other, you can take advantage of the tipping and rocking and rotation and you can often trick them in, but can not do so in this case. You must secure comparatively parallel relations of the adjacent surfaces.

I have found that the best impressions, the most accurate, are obtainable by taking these impressions in sections. You can take them either with modeling compound or complaster. Assuming, then, that we take them in complaster, it being the easier of the two materials to work with, we take a tray that covers the lingual and occlusal surfaces of these teeth. We do not care specially about the buccal surfaces now. We first want the lingual and occlusal surfaces of these teeth.

Put the material in so you can bend the wings to conform to the curvature and irregularities of the teeth. The impression material is carried down and forced up against the lingual and occlusal surfaces of the teeth. Take an instrument with a flat blade and cut away the excess back to the center of the tooth so you can withdraw the impression linguo-occlusally. If you push it out directly, at right angles, you probably will break off part of the impression. But if you withdraw it toward the occlusal and lingual at the same time it will come out easily.

Study the draw of the case from the mouth and design your impression or take your impression with reference to the draw so that when you cut away the excess that comes out bucally, it will not interfere with your impression lingually.

The major half of the impression is in the lingual impression. When removed trim and soap, or oil, and put it back in position, and with a similar tray take the buccal surfaces.

Make the models of inlay investment compound the same as for the single cast clasp. Cast these all in one piece for the reason that we have a double abutment and a clasp at both ends of the short space and depend largely upon these large, accurately-fitting clasps for masticatory resistance. We are not depending on the saddle. These short bridges that are made where we depend on the saddle area for resistance to the masticatory forces are of little value, so that the rule should be that in smaller cases where we have small saddles we should have good, heavy, large clasps in order that they be of any degree of comfort and usefulness to the patient. Then we are not particularly concerned whether we have the compressed relation of the saddle area in its relation to the clasps. We are going to depend on the clasps largely to carry the load and then, too, it is a difficult matter to make these casts in sections by casting the saddles and the clasps separately, and then assembling them. You have such a small saddle, working in such close quarters, it is difficult to keep it in place in its proper position while you are taking an impression of the saddle and the clasp. To simplify the work and make a very much more accurate and better appliance, make the casting all in one piece.

After securing the model, cut away all other teeth so that only the two teeth we are going to clasp remain on the model. We almost always have in these cases an under-cut that must be filled in so that our casting will not interfere with the removal and placement of the appliance. In order that we may fill that and cast over it, it is necessary that we take an instrument and cut a hole in our model of sufficient depth that we can build it up with some of the same material.

I refer to making this under-cut or this hole into your model, digging out so that when you place this addition it will be mechanically retained in position; otherwise, when you dry out your cast, this piece of investment compound is very liable to fall out of place unless it is mechanically retained in its position. In order to make sure, we dig a hole and then plaster up and parallel these proximating surfaces, at least if you want to be real sure, make the relation so they diverge rather than converge from the gingiva. We wax that so that we have the clasp on the bicuspid and the saddle and the clasp on the molar all in one continuous pattern.

In order to take advantage of gravity in casting, I know some say that this is not necessary, that you can cast up-hill as well as down, but I believe a good deal in the force of gravity, and I believe in all cases the wax pattern should be below the sprue attachment and I have been casting full plates and all kinds of plates and saddles with one sprue and with a very uniform success by taking advantage of gravity with a very simple device. There is no need of complicated machinery; no need of complicating this work. I am trying to make this work as simple as possible, to simplify the equipment, and I know that many will laugh at the outfit we are using but I am willing to take a chance on the results.

In order to take advantage of that principle of gravity I extend the wax entirely around the molar and make a complete ring around the molar and attach the sprue to the distal. That makes a complete ring around the molar, but we saw off the casting at this point which gives us an open ring type of clasp. That is merely done to facilitate the casting. We can just as well cast a base for a crown at the same time, or wax up and fit in the crown, casting the base for the crown and the whole appliance, so that all you have to do is cement your crown on and it is finished. This is a very simple, direct, accurate means of making these small bridges.—*Dental Summary,* December, page 899.

